# Cognition enhancing effect of rosemary (*Rosmarinus officinalis* L.) in lab animal studies: a systematic review and meta-analysis

**DOI:** 10.1590/1414-431X2021e11593

**Published:** 2022-02-09

**Authors:** S.M. Hussain, A.F. Syeda, M. Alshammari, S. Alnasser, N.D. Alenzi, S.T. Alanazi, K. Nandakumar

**Affiliations:** 1 Department of Pharmacy, City University College of Ajman, Ajman, United Arab Emirates; 2 Department of Pharmaceutics, Unaizah College of Pharmacy, Qassim University, Unaizah, Saudi Arabia; 3 Department of Pharmacy Practice, Unaizah College of Pharmacy, Qassim University, Unaizah, Saudi Arabia; 4 Department of Pharmacology, Unaizah College of Pharmacy, Qassim University, Unaizah, Saudi Arabia; 5 Saudi Food and Drug Authority, Riyadh, Saudi Arabia; 6 Clinical Laboratory Sciences, College of Applied Medical Sciences, King Saud University, Riyadh, Saudi Arabia; 7 Manipal College of Pharmaceutical Sciences, Manipal Academy of Higher Education, Manipal, India

**Keywords:** Rosemary, Cognition, Herbal, Alzheimer's disease, Rosmarinic acid

## Abstract

Patients with mild cognitive impairment eventually progress to Alzheimer's disease (AD) causing a strong impact on public health. *Rosmarinus officinalis* has long been known as the herb of remembrance and can be a potential cognition enhancer for AD. The aim of this review was to summarize the qualitative and quantitative aspects of *R. officinalis* and its active constituents in enhancing cognition. A structured search was conducted on Google Scholar and PubMed to find relevant studies that assessed the effect of *R. officinalis* extract or any of its active constituents on cognitive performance in animals. The following information was extracted from each study: 1) article information; 2) characteristics of study animals; 3) type of intervention: type, dose, duration, and frequency of administration of *R. officinalis*; and 4) type of outcome measure. Data were analyzed using Review Manager and meta-analysis was performed by computing the standardized mean difference. Twenty-three studies were selected for qualitative analysis and fifteen for meta-analysis. From the fifteen included papers, 22 with 35 comparisons were meta-analyzed. Effect sizes for intact and cognitively impaired animals were 1.19 (0.74, 1.64) and 0.57 (0.19, 0.96), indicating a positive effect on both groups. The subgroup analyses showed substantial unexplained heterogeneity among studies. Overall, *R. officinalis* improved cognitive outcomes in normal and impaired animals, and results were robust across species, type of extract, treatment duration, and type of memory. However, studies had a considerable amount of heterogeneity, and subgroup analyses failed to find any heterogeneity moderator.

## Introduction

Mild cognitive impairment is a deficit in memory and cognition with no physical limitation in daily activities. This impairment advances with age and is expected to increase in the future with an increasing older population worldwide. Patients with mild cognitive impairment frequently progress to dementia and Alzheimer's disease (AD), placing a heavy burden on the public health system (1-3). Hence, it is necessary to delay this progression for which there are many strategies including the use of cognitive enhancers (also referred as nootropics). Medications approved as cognitive enhancers for the treatment of AD include cholinesterase inhibitors (e.g., donepezil, rivastigmine, and galantamine) and the N-methyl-d-aspartic acid receptor antagonist (memantine) (4,5). Currently, these drugs are the mainstay of treatment, but their effectiveness is controversial, and each has its own set of adverse effects and limitations.

Plants have always been the most readily used resource for the treatment of diseases by humans. According to the World Health Organization (WHO), nearly 80% of the world's population relies on traditional medicine for primary health care needs and the potential of plants as a source of new drugs remains largely unexplored despite many advances (6,7). Interest in herbal medications as cognitive-enhancers is increasing with several promising compounds made available for the purpose, such as curcumin, *Ginkgo biloba*, *Bacopa monnieri*, *Hupericum perforatum*, *Salvia officinalis* (sage), huperzine A (*Lycopodium serratum*), and ginseng (8-13). These herbs have shown to be promising cognition enhancers, especially for the treatment of AD due to their cognitive benefits and, more importantly, for their mechanisms of action that address the fundamental pathophysiology of the disease in various preclinical and clinical trials.

**Figure 1:**
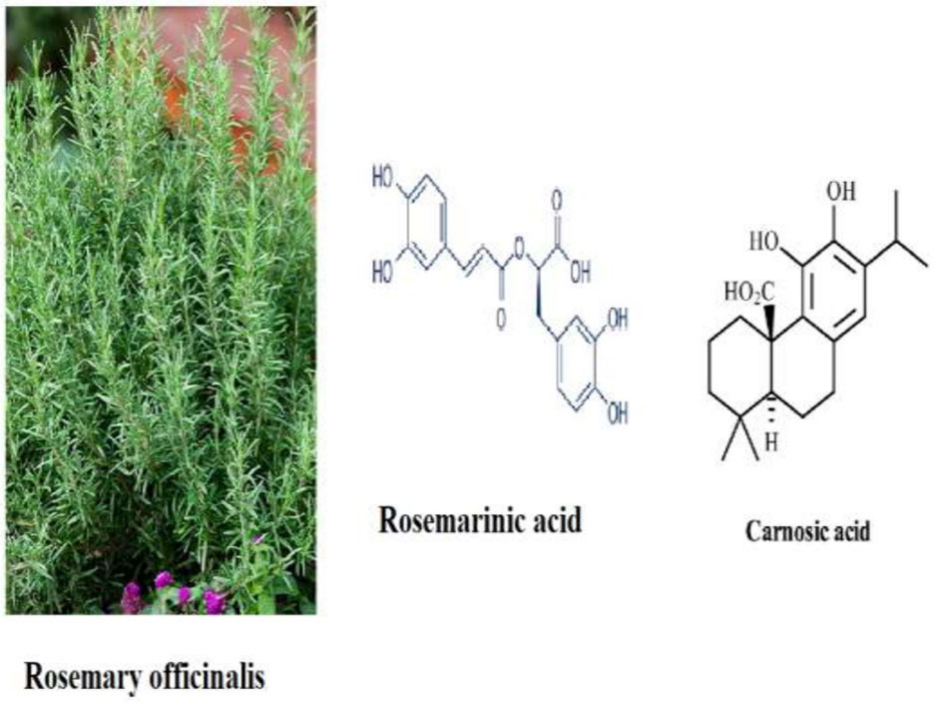
Rosemary plant and structures of its chemical constituents.

*Rosmarinus officinalis* Linn. (Lamiaceae), (*R. officinalis*, rosemary) is an aromatic plant (Figure 1) common in the Mediterranean region. It is the most cultivated culinary herb cultivated in the world. Its fresh and dried leaves are used in cooking or as herbal tea because of their characteristic fragrance. *R. officinalis* has been documented for its biological activities, such as antibacterial, anticancer, antidiabetic, anti-inflammatory and antinociceptive, antioxidant, antithrombotic, antiulcerative, cognitive deficit enhancement, antidiuretic, and hepatoprotective effects (14-25). *R. officinalis* has long been especially regarded as the herb of remembrance and occupies a special place in folk medicine (26,27).

Phenolic diterpenes, triterpenes, phenolic acids, such as carnosic acid (CA), carnosol, rosmanol, ursolic acid, betulinic acid, and rosmarinic acid (RA), and nepitrin are pharmacologically active constituents identified in *R. officinalis.* Among the isolated phenolic compounds in *R. officinalis*, CA and RA have been shown to have the most prevalent pharmacological effects and to interact with multiple molecular targets (26-31). The potential effects of *R. officinalis* in cognitive disorders and their influence on cognitive function have not yet been systematically reviewed. Therefore, the purpose of the present study is to summarize the qualitative and quantitative aspects of *R. officinalis* and its active components for improving cognition in preclinical studies and to identify their underlying mechanisms.

## Material and Methods

### Search strategy

The present review and meta-analysis are based on published results of animal studies on the effects of *R. officinalis* on cognitive performance, which were identified via a structured search on Google Scholar and PubMed to find relevant studies (last search run on June 2020). In Google Scholar, the following search terms were used: “*R. officinalis*”, “*R. officinalis* extract”, “rodent”, “animal”, and one of the following: “nootropic”, “cognitive enhancing”, “cognitive enhancers”, “memory enhancing”, or “memory enhancement”, “memory and learning”. For PubMed, the following key words (MeSH) were used with *R. officinalis*, rodent, and lab studies as the main search concepts: “nootropic”, “cognitive enhancing”, “cognitive enhancers”, “memory enhancing”, or “memory enhancement”, “rmemory and learning”. We also searched reviews to find additional relevant studies. No limits were applied to either date or language of the published studies.

### Inclusion criteria

The broad eligibility criteria were studies that examined the effect of *R. officinalis vs* a suitable control in healthy intact or cognitively impaired animals, and with learning and memory indices of task performance as outcomes. Thus, in conjunction with the above three broad criteria, studies were included based on the first criterion if they met the following: 1) random assignment of animals to groups; 2) animal groups with at least one healthy intact control group treated with vehicle and a healthy intact rodent group treated with *R. officinalis*; and 3) use of recognized test to measure learning and memory response to treatment. Studies were included based on the second criterion if they met the following: 1) any dosage of *R. officinalis* administered for any duration *vs* healthy intact control or cognitively impaired control; and 2) clear method of extraction or isolation of active constituent of *R. officinalis*. Studies were included for a third criterion if they measured the duration or speed of learning performance and memory task by animals.

### Exclusion criteria

**Figure 2:**
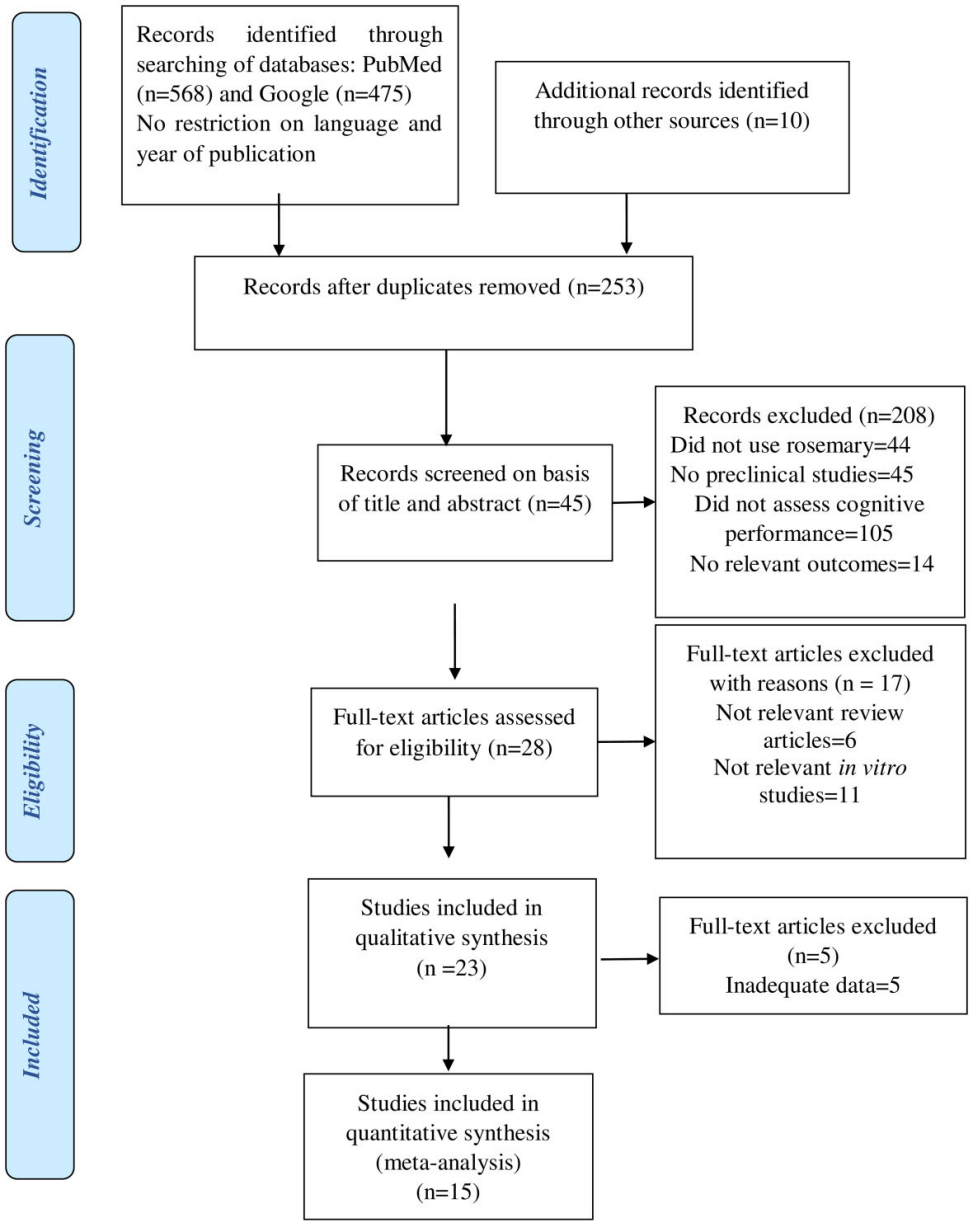
Flow chart of study selection process. The number of studies in each phase is indicated within parentheses.

Review articles, case reports, clinical studies, studies involving tasks that focused on other behavioral effects of *R. officinalis* on rodents were all excluded. The flow of information from identification to inclusion of studies is summarized in Figure 2.

### Screening and selection of studies

Study selection and systematic review were performed in accordance with the Preferred Reporting Items for Systematic Reviews and Meta-Analyses (PRISMA) statement (32).

Studies were included in the systematic review and meta-analysis if they fulfilled all of the following criteria: 1) the study assessed the effect of *R. officinalis* extract or any of its active constituents on cognitive performance; 2) the study was performed in animals *in vivo*; and 3) the study was an original full paper and presented unique data. Clinical studies and *in vitro* studies were excluded. Titles and abstracts of studies retrieved using the search strategy were screened and the full text of potentially eligible studies was retrieved and independently assessed for eligibility by two review authors. Any disagreement between the two review authors over the eligibility of specific studies was resolved through discussion with a third review author (Figure 2).

### Data extraction

We used a standard protocol to extract relevant data. Two authors independently extracted the following information from each included study: 1) article information (author and publication year); 2) characteristics of study animals (species, age, weight); 3) type of intervention; type, dose, duration, and frequency of administration of *R. officinalis*; and 4) type of outcome measure (task name and all indices of task performance that were used to assess cognition function). The characteristics of the studies are shown in Supplementary Table S1. Data used were group averages, standard deviation (SD) or standard error (SE), and number of animals per group (n). If SE was reported, this was converted to SD for meta-analysis. If a study conducted experiments with different tools, the data were extracted separately and treated as independent experiments for moderator analyses and combined to obtain a single effect size (standardized mean difference, SMD) for the respective study after suitable adjustment of its effect direction with an algorithmic sign. If outcomes were measured at several time points, we used only the results obtained on the day after the first measurement. When data were represented graphically, they were measured using web image analysis software (Web Plot Digitizer 4.2; automeris.io) (33). Eligibility assessment was performed independently in a standardized manner by two reviewers. Disagreements between reviewers, if any, were resolved by consensus.

### Statistical analysis

Data were analyzed using Review Manager 5.4 (RevMan, Cochran Collaboration). Meta-analysis was performed for the outcome measures (as a continuous outcome variable) on all relevant tasks reported by the included papers by computing the SMD (Hedges' g) of the treatment effect on intact and cognitively impaired rodent groups. Effect size of each study and pooled effect sizes and their confidence intervals (CI) were calculated by weighting using their inverse variance. Random effect models and I-squared (I^2^) test were used to quantitatively assess the impact of anticipated study heterogeneity on the results of the meta-analysis. P value <0.1 and I^2^ value >50% were considered statistically significant. A forest plot was generated to depict the SMD and 95%CI for each study as well as for the pooled value. Subgroup analyses were performed to assess the influence of moderators on *R. officinalis* efficacy, as well as to explore possible causes for heterogeneity. For subgroup analyses, multiple tasks of a study were entered as independent studies. The moderators used were duration of *R. officinalis* administration (acute/chronic), type of *R. officinalis* (whole extract/active constituent), type of memory predominantly assessed (reference memory/recognition memory), species of animal used (rat/mouse), and condition of animal (normal intact/cognitively impaired). Each of these moderators were individually entered into separate forest plots as a subgroup to assess subgroup interactions. A forest plot was built to depict the final effect of *R. officinalis* on normal intact and cognitively impaired animals using the SMD (95%CI) for each study (weighted sum of multiple tasks) as well as the pooled mean difference by combining all studies. In studies in which multiple tasks were conducted, the weighted score was divided by the number of tasks within a study.

### Qualitative data of the effects of *R. officinalis*

The papers included in the quantitative study and other relevant studies were reviewed to gather data on the various extracts, active constituents of *R. officinalis*, and their effects in improving cognitive performance in preclinical settings.

Karim et al. (34) isolated nepitrin from *R. officinalis* (doses of 50, 100, and 200 mg/kg, *po*, 60 min before the tests) to investigate its antiamnesic effect in Swiss male albino mice using the Y-maze and NORT (novel object recognition) tests. In the Y-maze test, it produced a significant (P<0.01) dose-dependent decrease in entries in the same arm and an increase in alternating arms. Similarly, in the NORT test, nepitrin-treated mice spent a longer time investigating the novel object indicating an increased discrimination compared to familiar objects. In *in vitro* studies, nepitrin showed concentration-dependent anticholinesterase and antioxidant activities.

Ozarowski et al. (35) used ethanolic extract of leaves of *R. officinalis* (at a dose of 200 mg/kg) and isolated rosmarinic acid (RA, 10 mg/kg) on male six-week-old Wistar rats, which produced no significant effect but overcame the effects of pretreated scopolamine assessed using the passive avoidance (PA) and NORT tests. In isolated brain regions of treated animals, RE and RA showed inhibition of AChE and enhancement of BuChE activities in the frontal cortex and hippocampus.

Song et al. (36) used commercial extracts of *R. officinalis* containing 20% carnosic acid (at 40, 80, and 160 mg/kg) on mild traumatic brain injury (mTBI)-induced adult male Sprague-Dawley rats in which the extract was administered *po* for 16 days during training and testing for 1-7 days post-injury. In the Morris water maze (MWM) test, the treatment restored the spatial learning and memory deficits induced by mTBI. Tissue analysis also revealed that the *R. officinalis* treatment reduced the mTB-induced degeneration of neurons, astrocytosis, oxidative stress, and inflammatory cytokines in the hippocampus.

Ferlemi et al. (37) used the infusion of *R. officinalis* leaves (administered 2% w/v per day for 4 weeks) in adult male Balb-c mice using the PA test. Administration of *R. officinalis* did not produce significant changes in latency time. However, tissue analysis of the brain and liver showed significantly decreased AChE activity.

Zanella et al. (38) used hydroalcoholic extract of *R. officinalis* (at doses of 10, 150, and 300 mg/kg) in adult Swiss male mice and performed experiments using social recognition (SR), MWM, and PA tasks. The treatment with 150 and 300 mg/kg of *R. officinalis* improved the acquisition phase of learning in SR but in MWM, no significant effect was observed. However, in PA, *R. officinalis* at 150 mg/kg improved long-term memory in the consolidation phase of learning.

Rasoolijazi et al. (39) used *R. officinalis* extract (containing 40% carnosic acid) at doses of 50, 100, and 200 mg·kg^-1^·day^-1^ *po* for a period of 12 weeks in adult male Wistar rats. *R. officinalis* at 100 mg/kg significantly (P<0.05) improved spatial memory in the MWM, and isolated brain tissue analysis revealed a significantly increased activity of antioxidant enzymes in the hippocampus.

Farr et al. (40) evaluated the effects *R. officinalis* and spearmint extracts containing carnosic acid (60% or 10%) and rosmarinic acid (5%), respectively, using the specially inbred SAMP8 mice model of accelerated aging. Three dose levels were selected (32, 16, 1.6 mg/kg). After treatment for 90 days, the mice were tested in 3 different tests: T-maze foot shock avoidance, NORT, and lever press tests. *R. officinalis* with 60% CA improved acquisition and retention in all three tests whereas *R. officinalis* with 10% CA improved only acquisition. On the other hand, spearmint extract with 5% RA improved both acquisition and retention in the T-maze foot shock avoidance and lever press tests, respectively. In brain tissue analysis, all the extracts reduced the 4-hydroxynonenal (HNE) in the cortex significantly. There was also a significant reduction in protein carbonyls in the hippocampus by both *R. officinalis* with 10% CA and spearmint extract with 5% RA.

Rasoolijazi et al. (41) injected beta-amyloid (Aβ (1-40)) by stereotaxic surgery into the Ca1 region of the hippocampus of rats, and the administration of CA (10 mg/kg, *ip*) was done before and after surgery. PA and Y-maze tests were conducted to observe the effect of Aβ and CA treatment on learning and memory behavior. CA prevented the Aβ-induced deficiencies in step-down latency and spontaneous alternation behavior scores in the PA and Y-maze, respectively. Tissue analysis also revealed that CA reduced the degeneration of hippocampal neurons.

Hosseinzadeh et al. (42) studied the effect of essential oil of *R. officinalis* at doses of 125-250 mg/kg for 5 consecutive days on male Wistar rats. The MWM test was used to assess the effects on scopolamine-induced learning deficits and normal rats. The essential oil decreased the latency time to find the platform in both normal and scopolamine-induced rats.

Capatina et al. (43) studied the effect of *R. officinalis* essential oil (25, 150, and 300 µL/L) administered by immersion to scopolamine-induced deficient zebra fish once daily for eight days. The test used was a modified Y-maze for zebra fish where treated fish showed an increased time spent in the novel arm of the Y-maze indicating a cognitive-enhancing action and abolished the scopolamine-induced AChE alteration in brain autopsy compared to untreated control fish.

Lee et al. (44) investigated the effect of RA (0.25 mg·kg^-1^·day^-1^, *po*) for 14 days in Aβ 25-35-induced deficits in male ICR mice using the T-maze, NORT, and MWA tests. RA significantly enhanced alternation movements, object discrimination, and decreased latency to reach the platform in the T-maze, NORT, and MWM tests, respectively. Furthermore, RA significantly decreased the levels of nitric oxide (NO) and malondialdehyde (MDA) in the brain, kidney, and liver indicating a cognitive improvement.

Hasanein et al. (45) studied the effect of RA in streptozocin-induced diabetic and non-diabetic adult male Wistar rats. Diabetes-induced deficits in acquisition and retrieval processes were examined after 30 days of treatment with RA using the PA test where it showed increased step-down latency (enhanced cognition). The treatment also enhanced antioxidant enzymes superoxide dismutase and catalase in blood.

Alkam et al. (46) used Aβ 25-35-induced male ICR mice who were treated with RA (0.05, 0.25, 1, 2, and 4 mg·kg^-1^·day^-1^, *ip*) for 14 days and increased spontaneous alternation behavior and increased novel discriminatory exploration were observed in the Y-maze and NORT tasks, respectively. In *in vitro* studies, RA prevented Aβ 25-35-induced nitration of proteins, indicating a scavenging of ONOO effect, demonstrating the memory protective and enhancing effect of RA.

Pereira et al. (47) used adult Wistar rats to investigate the effect of RA (1, 2, 4, or 8 mg/kg, *ip*) using the PA apparatus. RA (2 and 4 mg/kg) caused a significant increase in step-down latency. The brain images of treated rats showed no significant DNA damage by RA.

Park et al. (48) found that RA inhibited prolyl oligopeptidase (POP) activity with an IC(50) of 63.7 µM and chronic RA treatment increased the platform crossings in Morris water maze paradigm. Their findings suggested that RA may have a cognitive-enhancing effect via POP inhibition.

Depeursinge et al. (49) demonstrated the ability of RA to enhance the cognitive effect in MWM test in adult male ICR mice following acute and subchronic treatment. It also caused the inhibition of prolyl oligopeptidase (POP) in the brain.

Kosaka and Yokoi (31) carried out the extraction of dried leaves of *R. officinalis* to prepare aqueous and alcoholic extracts. From the alcoholic extract, they isolated CA and carnosol through column chromatography and the extracts were tested on T98G human glioblastoma cells where an enhanced production of nerve growth factor (NGF) was found. CA was the most efficient and effective among them.

Orhan et al. (50) prepared various extracts and the essential oil of Turkish *R. officinalis* and tested for AChE and BChE inhibitory activities. The essential oil significantly inhibited AChE and BChE, and the other extracts did not produce a significant inhibition. However, RA, from the methanol extract of *R. officinalis,* showed a remarkable BChE-inhibitory effect.

Vladimir-Knezevic et al. (51) conducted a comparative study of a large number of lamiaceae medicinal plants containing RA, including *R. officinalis*. RA showed a strong inhibitory effect for AChE. All the tested extracts also demonstrated moderate to strong antioxidant activities.

Hase et al. (52) proposed a new mechanism for the inhibition of Aβ. Aggregation by RA polyphenols and monoamines is via an o-quinone structure, which specifically binds to Aβ and prevents further aggregation. Dopamine (DA) in its oxidation state transforms to o-quinone structure and interferes with Aβ aggregation. RA suppresses Aβ accumulation in mouse brain by increasing the concentration of monoamine including DA.

Cornejo et al. (53) demonstrated that RA is the most active compound that inhibits tau fibrillation and prevents β-sheet assembly.

El Omri et al. (54) carried out tests in PC12 cells where various extracts and fractions of *R. officinalis* caused a dose-dependent increase in AChE activity. CA and RA induced differentiation and improved total choline level and ACh synthesis in PC12 cells. Neurotrophic effects were also shown in PC12 cells leading to attenuated atrophy of cholinergic neurons, which would result in an enhancement of memory, attention, and impaired behavior.

## Results

### Study identification and selection

The electronic search retrieved 568 records from PubMed, 475 from Google Scholar, and 10 from other sources. After eliminating duplicates, 253 were left. Following the screening of titles and abstracts, 45 were retained for the full text evaluation. Twenty-eight papers met our inclusion requirements, out of which twenty-one (34-54) focusing on the cognitive impact of *R. officinalis* on animal models were included in the qualitative study and 15 from those were further chosen for quantitative analysis (34-48).

### Study characteristics

The characteristics of the included studies are summarized in Supplementary Table S1. There was a large variation in the characteristics of selected studies with respect to study design, animal models, outcome measures, and study animals. The studies were conducted between 2004 and 2020 in different countries and had in total 35 comparisons; one study was performed with zebra fish as the study animal, 11 studies used rats, and 10 studies were performed on mice. There were 488 study animals as participants: 232 rats, 236 mice, and 20 zebra fish. Among the studies, 13 had normal healthy animals and 9 had cognitively impaired animals. Out of 15 papers, three papers reported only intact animals that were cognitively impaired by chemical or physical means, 6 papers reported normal animals, and 6 reported both cognitively impaired animals and normal animals. There were 298 normal animals and 190 cognitively impaired animals. The common method used to induce impairment in animal cognition was scopolamine (n=5), Aβ protein (n=3), brain injury (n=1), and STZ (n=1). All were published in the English language except one that was published in Persian [abstract available in English (42)] and was translated. The duration of *R. officinalis* administration ranged from 30 min (n=4), to one week (n=3), to several weeks (n=8) before the experiment. The common form of the *R. officinalis* used in the experiments was the whole extract (n=6), any of the active constituents (n=8) of the plant, and some studies employed both (n=1). The active constituents that were explored in these studies were RA (n=6), CA (n=2), and nepitrin (n=1). The common route of administration was oral (n=10), intraperitoneal (n=4), and by immersion (n=1). All the included studies assessed change in cognitive performance between control and treated groups as an index of cognitive function. For measuring the cognitive function, different tests and tools were used including MWM (n=5), passive and active avoidance paradigms (n=6), T- and Y-mazes (n=6), and social (n=1) and novel object recognition tests (n=4). The types of memory that were assessed using different tools (n=35) were reference memory (n=26) and recognition memory (n=9). Some studies performed multiple tasks (n=6) to assess cognitive functions. The common index used to assess performance was latency to reach a target or escape an aversive stimulus, time spent in target area, and time spent investigating a novel object (Supplementary Table S1).

### Methodological quality of included studies

We assessed the quality of the included studies using the 10-point rating system checklist of the Collaborative Approach to Meta-Analysis and Review of Animal Data from Experimental Studies (CAMARADES) applicable to preclinical studies (55). One point was given for each quality criterion. The quality of all studies was assessed independently by two reviewers. As shown in Supplementary Table S2, the quality score of the included studies ranged from 7 to 9 of a possible total of 10 points. The majority of the studies reported the randomization of animals into treatment groups but did not mention the method of randomization. All studies were published in peer-reviewed journals and stated the potential conflict of interests or funding sources. However, none of the studies reported blinded assessment of outcome measures and none of the studies described the method for calculating the sample size.

### Meta-analysis

**Figure 3:**
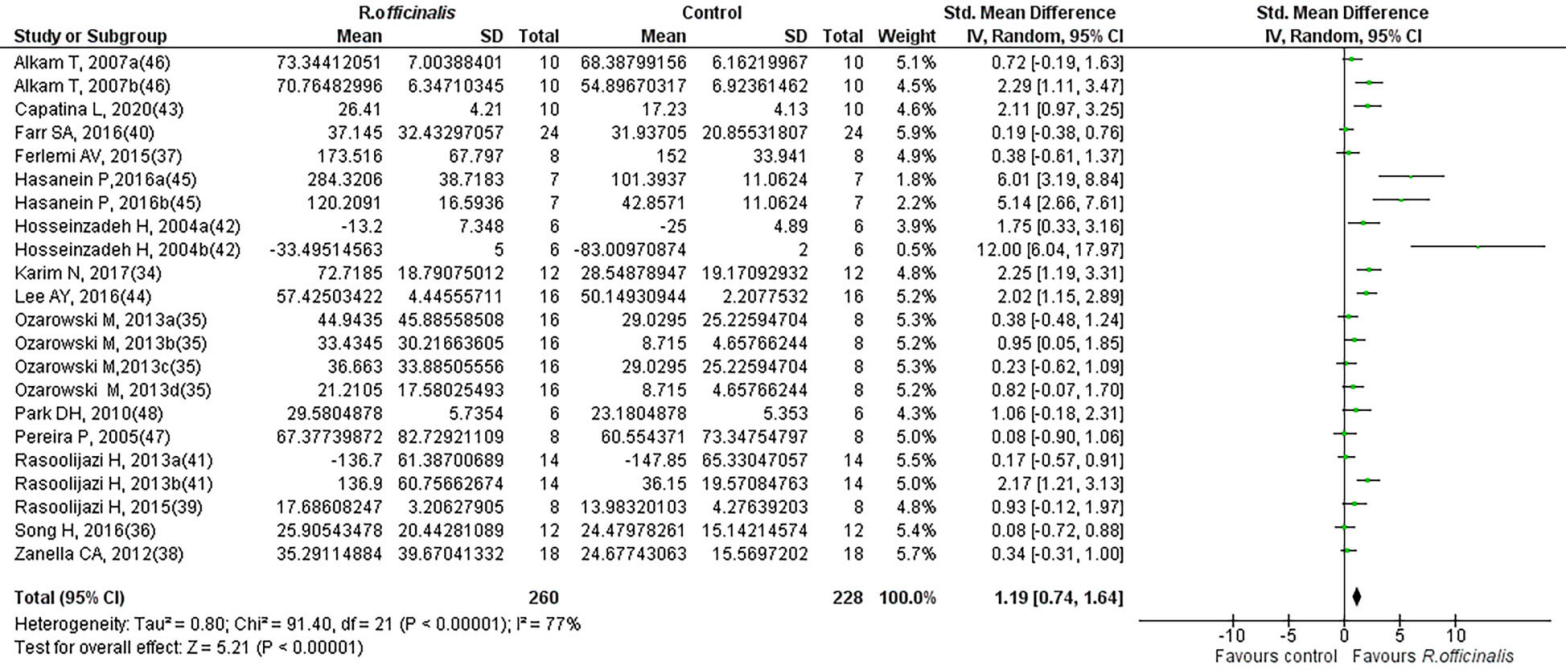
Forest plot of the effect of *R. officinalis* on cognitive performance in lab animals.

**Figure 4:**
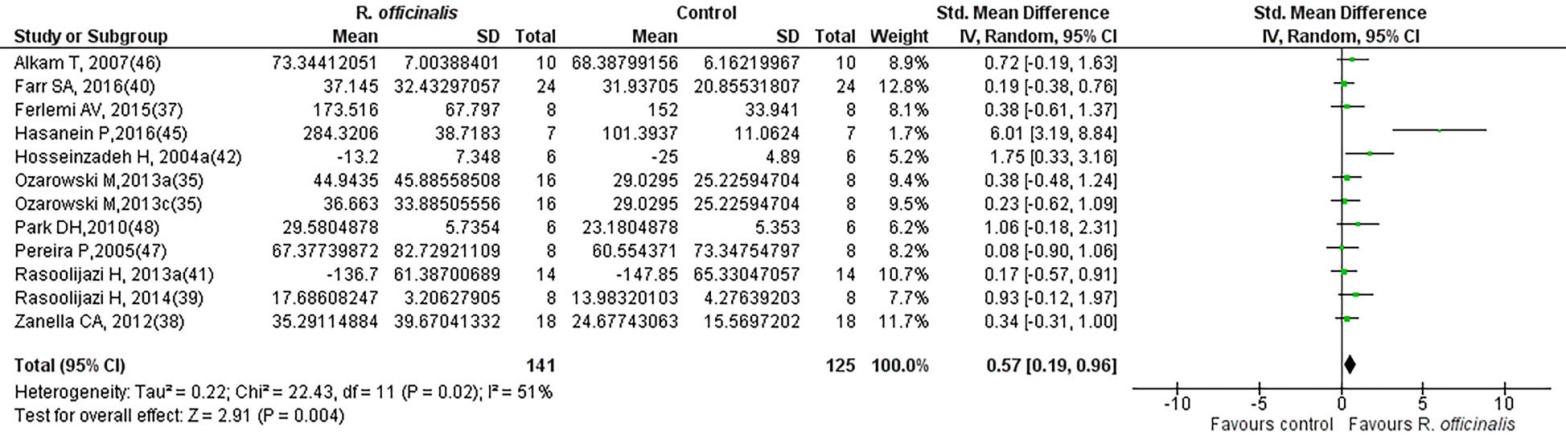
Forest plot of the effect of *R. officinalis* on cognitive performance in normal animals.

**Figure 5:**
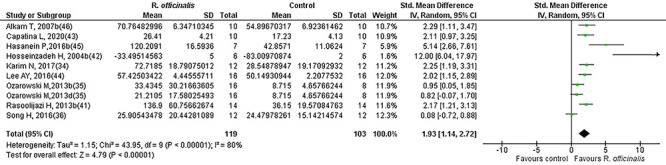
Forest plot of the effect of *R. officinalis* on cognitive performance in cognitively impaired animals.

Results for cognitive performance are summarized in Table 1 and Figure 3. The random effect model with inverse variance was adopted to generate forest plots of effect sizes with the Hedges' g test. Hedges' g was used due to the heterogeneous nature of preclinical studies and to account for anticipated biases. The analysis included the data of 248 normal control animals and 280 animals that underwent treatment with *R. officinalis* (maximal dose only). Figure 3 illustrates the effect size (SMD, Hedges' g) and 95%CI for each of the 22 comparisons from the 15 selected studies. A large positive effect of *R. officinalis* for normal rodents' performance (mean g and 95%CI: 1.19 [0.74, 1.64]) was found across studies, which was statistically significant (Z=5.21, P<0.00001), indicating memory enhancement among intact rodents due to administration of *R. officinalis*. A visual assessment of the results suggested between-study variability and the treatment effect point estimates of the majority of the studies were on the same side of the line of null effect but did not overlap, indicating a difference in treatment effect magnitude among studies. The CI for treatment effect of each study (horizontal lines) overlapped, but the upper and lower limits of the CI did not consistently line up on a vertical axis, indicating differences in the population treatment effect among studies, suggesting the presence of significant heterogeneity (chi^2^=91.40, df=21, P=000001, I^2^=77%). Figures 4 and 5 represent the forest plots of effect sizes for the *R. officinalis* effect on normal and cognitively impaired animals, respectively. Figure 4 shows the results for 12 studies that included the data for 125 normal control animals and 141 animals that underwent treatment with *R. officinalis* (maximal dose only). The overall estimate indicates a positive effect of *R. officinalis* on the performance of normal control animals (mean *g* and 95%CI: 0.57 [0.19, 0.96], P<0.004]. Similarly, Figure 5 shows the results of 10 studies that included the data of 103 cognitively impaired animals and 119 cognitively impaired animals that underwent treatment with *R. officinalis* (maximal dose only). The overall estimate for the treated impaired animals was stronger (mean *g* and 95%CI: 1.93 [1.14, 2.72], Z=4.79, P<0.0001) than for the normal treated animals.

**Table 1:** Comparison of subgroup estimates (Hedges g, 95%CI) and overall effect.

Subgroup	Studies	Participants	Effect Estimate (SMD)	Overall effect Z value (P)
Animal condition	22	488	1.19 [0.74, 1.64]	5.21 (P<0.00001)
Intact	12	266	0.57 [0.19, 0.96]	2.91 (P=0.004)
Impaired	10	222	1.93 [1.14, 2.72]	4.79 (P<0.0001)
Animal species	21#	468#	1.14 [0.69, 1.60]	4.93 (P<0.00001)
Rat	11	232	0.80 [0.25, 1.34]	2.88 (P=0.004)
Mouse	10	236	1.58 [0.81, 2.35]	4.02 (P<0.0001)
Type of drug	22	488	1.19 [0.74, 1.64]	5.21 (P<0.00001)
Extract	10	240	0.61 [0.25, 0.96]	3.36 (P=0.0008)
Active	12	248	1.86 [1.05, 2.67]	4.51 (P<0.00001)
Duration of treatment	22	488	1.19 [0.74, 1.64]	5.21 (P<0.00001)
Acute	4	76	1.35 [-0.24, 2.94]	1.66 (P=0.10)
Chronic	18	412	1.21 [0.74, 1.68]	5.02 (P<0.00001)
Type of memory assessed	35*	488	1.53 [1.04, 2.03]	6.11 (P<0.00001)
Working	26	298	1.74 [1.14, 2.33]	5.71 (P<0.00001)
Recognition	9	190	1.06 [0.15, 1.97]	2.28 (P=0.02)

# The only study performed with zebra fish was omitted in the subgroup analysis.

* All the individual experiments performed to assess memory were entered in the subgroup analysis. SMD: standard mean difference.

### Stratified analysis

As the effect estimates in the present study are the outcome of the combination of various subsets of data in individual studies, the features or characteristics that can influence the effect size might be concealed. In this respect, stratified meta-analyses were conducted to investigate the heterogeneity of the data and its impact on the overall estimate of the *R. officinalis* efficacy on cognitive improvement in normal intact and cognitively impaired animals. Subgroups were created based on study animals (impaired *vs* normal intact), species used (rat *vs* mouse), memory assessed (reference memory *vs* recognition), extract used (whole extract *vs* active constituent), and duration of treatment (acute *vs* chronic). The treatment effect could be verified for all subgroups, which showed a beneficial effect of *R. officinalis* both for intact normal rodents and cognitively impaired rodents (intact *vs* impaired: *Z=*5.21 (P<0.001) (Table 1). Significant effects were observed for the other subgroup analyses: rat *vs* mouse (Z=4.93, P<0.001); extract *vs* active constituent, (Z=5.21, P<0.001); and reference memory *vs* recognition memory (Z=6.11, P<0.001). The difference for duration of treatment subgroup was non-significant (Z=1.66, P=0.10) (Table 1). However, the results could be due to the limited number of studies using a chronic treatment period (4 *vs* 18).

Table 2 shows the subgroup analyses for heterogeneity and group differences by chi^2^ and I^2^ methods. A statistically significant subgroup effect was found for normal *vs* impaired (P=0.002), meaning that the animal condition significantly modified the effect of *R. officinalis* treatment compared to control. The *R. officinalis* treatment had a significant effect for both normal and cognitively impaired animals, although the treatment effect was greater for cognitively impaired than normal; therefore, the subgroup effect was quantitative. A sufficient number of studies and participants were included in each subgroup, so the covariate distribution was not a problem for this subgroup analysis. However, there was a substantial unexplained heterogeneity among the studies within each of these subgroups (normal: I^2^=51%; cognitively impaired: I^2^=80%). Similarly, the heterogeneity study for type of extract yielded statistically significant subgroup effects in extract *vs* active constituent (P=0.005). The heterogeneity was non-significant for type of animal species, type of memory assessed, and duration of treatment of *R. officinalis* (Table 2).

**Table 2:** Subgroup analyses for heterogeneity and group differences (chi^2^ and I^2^ tests).

Subgroup	Heterogeneity chi^2^, df (P), I^2^	Subgroup differences chi^2^ (P), I^2^
Animal condition	91.40, 21 (P<0.00001), 77%	9.21, (P=0.002), 89.1%
Intact	22.43, 11 (P=0.02), 51%	
Impaired	43.95, 9 (P<0.00001), 80%	
Animal species#	86.57, 20 (P<0.00001), 77%	2.64, (P=0.10), 62.2%
Rat	32.76, 10 (P=0.0003), 69%	
Mouse	51.47, 9 (P<0.00001), 83%	
Type of drug	91.40, 21 (P<0.00001), 77%	7.72, (P=0.005), 87.0%
Extract	14.80, 9 (P=0.10), 39%	
Active	63.69, 11 (P<0.00001), 83%	
Duration of treatment	91.40, 21 (P<0.00001), 77%	0.03, (P=0.87), 0%
Acute	18.13, 3 (P=0.0004), 83%	
Chronic	71.55, 17 (P<0.00001), 76%	
Type of memory assessed*	168.6, 34 (P<0.00001), 80%	1.49, (P=0.22), 32.9%
Working	131.2, 25 (P<0.00001), 81%	
Recognition	37.39, 8 (P<0.00001), 79%	

# The only study performed with zebra fish was omitted in the subgroup analysis.

* All the individual experiments performed to assess memory were entered in the subgroup analysis.

Because of the high unexplained heterogeneity among studies, the validity of the treatment effect estimate for each subgroup was uncertain, as individual results about the benefits of *R. officinalis* on cognitive performance were inconsistent. Therefore, further investigations on the variability across studies are strongly recommended.

### Publication bias

**Figure 6:**
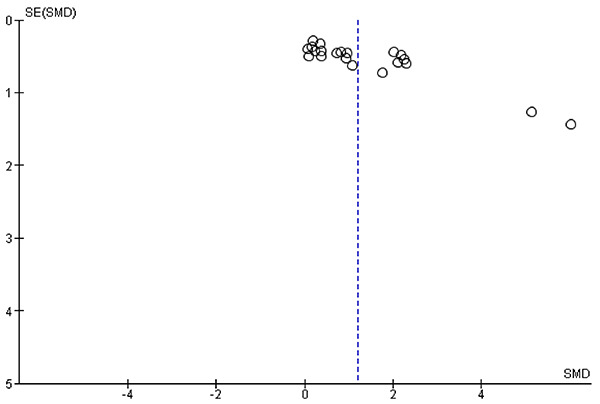
Funnel plot of experimental comparisons on normal rodents (maximal dosages only, different tasks shown separately). SMD, standardized mean difference.

Figure 6 shows the relationship between treatment effect and study precision by an asymmetric funnel plot with uneven distribution due to publication bias or differences between studies with higher and lower precision. Effect sizes were plotted against SE of the SMD, and this asymmetry may also be due to the use of an inappropriate effect measure, which warrants further investigation of possible causes. The funnel plot was based on all tasks involving study animals, and two outliers were found.

### Qualitative synthesis of included studies

The review of published literature on *R. officinalis* especially for cognitive performance in preclinical studies revealed that a wide variety of animal species were employed (Swiss male albino mice, Balb-c mice, ICR mice, SAMP8 mice, Wistar rats, Sprague Dawley rats, and wild-type short-fin strain zebra fish). *R. officinalis* was used in different forms such as extracts of whole plants, leaves, essential oil of the plants, isolated compounds, etc. To assess the cognitive enhancing potential of *R. officinalis*, various instruments and tasks were used: active and passive avoidance tests, MWM, Y-maze, T-maze, novel object recognition test, and social recognition paradigm. Similarly, *in vitro* tests employed a variety of experimental designs and cell lines, the most common being PC12 cells, Cacao cells, and T98G human glioblastoma cells. To assess the effect of *R. officinalis* in cognitively deficient animals, cognitive impairment was induced using various strategies including scopolamine, streptozotocin, Aβ, mild injury to brain, and genetically modified aging.

**Figure 7:**
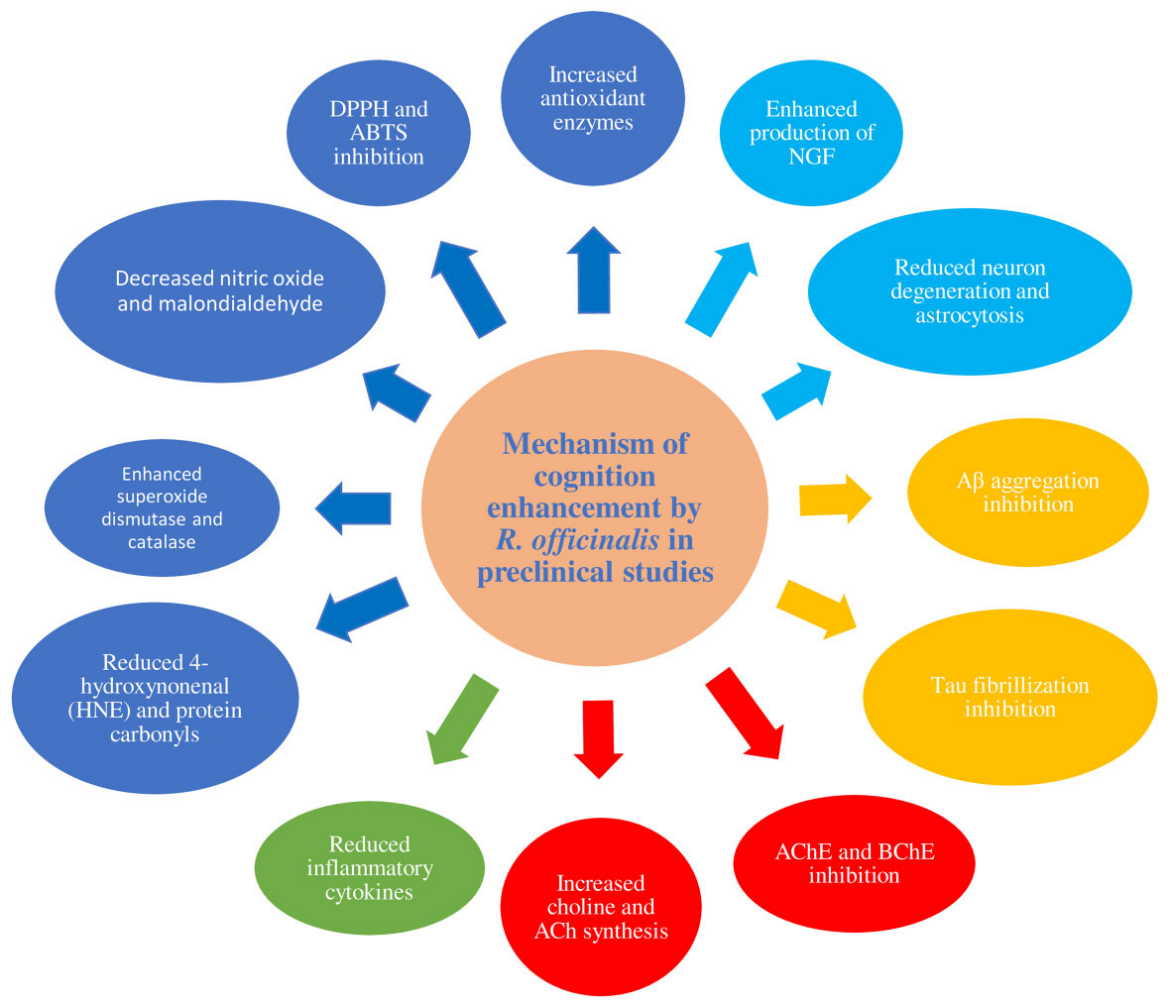
Mechanism of cognition enhancement by *R. officinalis* in preclinical studies.

The various studies assessed different aspects of learning and memory and the mechanisms of *R. officinalis* and its constituents in contributing towards a cognition enhancement were anticholinesterase, procholinergic, antioxidant, anti-amyloid, neuroprotective, and anti-inflammatory (Figure 7).

## Discussion

Systematic reviews and meta-analyses of animal studies help in gathering evidence for investigating the effects of experimental interventions before proceeding to clinical research involving humans. However, they also pose serious challenges due to the variability in the nature of experimental set-up, animal species, study characteristics, and lab design (56). The current study was the first to carry out a systematic review and meta-analysis to examine the efficacy of *R. officinalis* treatment for improving cognitive activities in animal models by assessing memory and learning. Overall, our study suggested that *R. officinalis* had the ability of improving cognitive outcomes in normal as well as cognitively impaired animals. Results were robust across species, type of extract, treatment duration, and type of memory.

The present meta-analytic study of *R. officinalis* revealed that it has large nootropic effects in the preclinical tests, although it was clear in the individual studies that the effect was more prominent in study animals with impaired cognitive performance than in normal animals.

Although these tests are designed to assess different components of memory or learning processes, in the present study they were broadly categorized into two types, one that assessed reference memory and another recognition memory (57-59). The experiments performed with MWM, active/passive avoidance paradigms, and T- and Y-mazes were considered to assess reference memory and the novel recognition tests and social recognition paradigms were considered to assess recognition memory. Interestingly, the cognition enhancing effect was observed in both tasks. This implies that the effect of *R. officinalis* on performance was mediated by the influence on broader learning and memory processes in treated animals. It is also noteworthy that the effect of *R. officinalis* was stronger in cognitively impaired animals than in normal intact animals. These findings demand further enquiry into the mechanistic and pharmacological characteristics of *R. officinalis* to clarify the effect on cognitive performance. Finally, the qualitative synthesis showed several pharmacological effects of *R. officinalis* and its active constituents in different models and experiments across studies. The mechanisms of action in exerting a nootropic effect also varied across studies as the essential oil of *R. officinalis* produced notable inhibitions of both AChE and BuChE enzymes and also restored the scopolamine-induced AChE alteration in the brain (44,45,50). CA was found to enhance production of NGF, total choline level, and Ach, attenuating the atrophy of cholinergic neurons. It also reduced the degeneration of hippocampal neurons against Aβ, reduced age-related brain tissue markers of oxidation, and increased the activity of antioxidant enzymes (31,54). Similarly, RA also improved total choline level and ACh synthesis, attenuating cholinergic neurons atrophy and inhibiting AChE activity. RA significantly decreased NO levels, MDA, and POP, and enhanced antioxidant enzymes superoxide dismutase and catalase without showing DNA damage in any brain parameter. Finally, RA inhibited Aβ aggregation by increasing concentration of monoamine, including DA, and inhibited tau fibrillation and prevention of Aβ-sheet assembly (35,53,60,61). In molecular docking studies, nepitrin also showed anticholinesterase and antioxidant activities. It occupied the same binding site and was found to form similar interactions to those formed by donepezil (anti-AChE agent) in the crystal structure of AChE, thereby confirming its AChE inhibitory activity (34). To conclude, *R. officinalis* and its associated chemical constituents have several mechanisms, such as anticholinesterase, procholinergic, antioxidant, anti-amyloid, neuroprotective, and anti-inflammatory, in contributing towards a cognition enhancement (Figure 7) (15-22,61).

There are also various clinical studies on the effects of *R. officinalis* on different aspects of memory. Pengelly et al. (62) reported that *R. officinalis* produced an increase in memory speed in the elderly population. Another study found that a combination of sage, *R. officinalis*, and lemon balm had significant effects on the improvement of verbal episodic memory in healthy people under 63 years of age (63). However, another study did not find a significant improvement in cognitive task performance in young adults with low energy (64). Another study reported that *R. officinalis* could boost prospective and retrospective memory, reduce anxiety and depression, and improve sleep quality in university students (65). Moss and Oliver (66) reported a positive correlation between plasma 1,8-cineole levels following aromatherapy with *R. officinalis* and cognitive performance. Another study assessed the efficacy of aromatherapy with essential oil of *R. officinalis* on cognition and on behavioral and psychological symptoms of dementia (BPSD) in patients with mild cognitive impairment and found aromatherapy to be safe and effective in this population (67).

### Limitations of the study

There are some limitations in our study. Firstly, animal models of cognitive deficit do not fully represent aspects of human cognitive function thereby limiting the translation of our results to humans. Secondly, potential selection publication bias is likely to exist despite our effort in identifying all the relevant studies. Our analysis did not take into account unpublished data, so the overall effect size could be overestimated. Subgroup analyses did not identify the moderators accounting for the heterogeneity among studies.

### Conclusions

This systematic review and meta-analysis indicated that administration of *R. officinalis* improved cognitive function in animal models of cognitive deficit and in normal intact animals. The outcomes may be used in the planning of clinical studies provided the included studies are robust enough to account for the heterogeneity observed. The cognitive benefits provided by *R. officinalis* and its mechanisms of action are in synchrony with the fundamental pathophysiology of cognitive deficit and the herb could be a potential treatment for Alzheimer's disease.

## Supplementary Material

Click to view [pdf].
